# Use of Optical and Radar Imagery for Crop Type Classification in Africa: A Review

**DOI:** 10.3390/s24113618

**Published:** 2024-06-03

**Authors:** Maryam Choukri, Ahmed Laamrani, Abdelghani Chehbouni

**Affiliations:** 1 Center for Remote Sensing Applications (CRSA), UM6P, Benguerir 43150, Morocco; ahmed.laamrani@um6p.ma (A.L.); abdelghani.chehbouni@um6p.ma (A.C.); 2 College Agriculture and Environmental Sciences, Mohammed VI Polytechnic University (UM6P), Benguerir 43150, Morocco; 3 Department of Geography, Environment & Geomatics, University of Guelph, Guelph, ON N1G 2W1, Canada

**Keywords:** earth observation, crop classification, SAR data, optical imagery, Africa

## Abstract

Multi-source remote sensing-derived information on crops contributes significantly to agricultural monitoring, assessment, and management. In Africa, some challenges (i.e., small-scale farming practices associated with diverse crop types and agricultural system complexity, and cloud coverage during the growing season) can imped agricultural monitoring using multi-source remote sensing. The combination of optical remote sensing and synthetic aperture radar (SAR) data has emerged as an opportune strategy for improving the precision and reliability of crop type mapping and monitoring. This work aims to conduct an extensive review of the challenges of agricultural monitoring and mapping in Africa in great detail as well as the current research progress of agricultural monitoring based on optical and Radar satellites. In this context optical data may provide high spatial resolution and detailed spectral information, which allows for the differentiation of different crop types based on their spectral signatures. However, synthetic aperture radar (SAR) satellites can provide important contributions given the ability of this technology to penetrate cloud cover, particularly in African tropical regions, as opposed to optical data. This review explores various combination techniques employed to integrate optical and SAR data for crop type classification and their applicability and limitations in the context of African countries. Furthermore, challenges are discussed in this review as well as and the limitations associated with optical and SAR data combination, such as the data availability, sensor compatibility, and the need for accurate ground truth data for model training and validation. This study also highlights the potential of advanced modelling (i.e., machine learning algorithms, such as support vector machines, random forests, and convolutional neural networks) in improving the accuracy and automation of crop type classification using combined data. Finally, this review concludes with future research directions and recommendations for utilizing optical and SAR data combination techniques in crop type classification for African agricultural systems. Furthermore, it emphasizes the importance of developing robust and scalable classification models that can accommodate the diversity of crop types, farming practices, and environmental conditions prevalent in Africa. Through the utilization of combined remote sensing technologies, informed decisions can be made to support sustainable agricultural practices, strengthen nutritional security, and contribute to the socioeconomic development of the continent.

## 1. Introduction

Global population is expected to attain 9.7 million by 2050 [1], with an estimated annual increase of 83 million individuals per year [2]. This is expected to cause a worldwide need for food that will shape future food security and conservation efforts [3]. These demographic challenges are even greater in Africa. To ensure a profitable agriculture in Africa and to make informed decisions, it is important to understand the state and trends of agricultural production. To this end, there is a need for crop mapping, yield assessment and monitoring based on Earth Observation (EO) data. To this end, there is a need for crop mapping, yield assessment and monitoring in terms of the EO data from varied platforms and advanced methodological approaches (i.e., classification, IA algorithms).

The ever-increasing availability of high resolution open-access EO data at both optimal spatial and temporal scales and powerful computing resources provides great opportunities for agricultural mapping applications. However, crop assessment and mapping in the context of African agriculture has been challenging, especially regarding data collecting, storing, and processing, and the requirement of the datasets to cover large geographic areas [4] to accomplish agricultural mapping applications.

Classifications based on single-source of optical satellite data were the focus of crop type monitoring for many years [5]. In addition, the use of one-source remote sensing data is often impeded by some challenges (i.e., the small size of the field, the complexity of cropping systems, the presence of cloud cover, particularly in African tropical regions). To this end, the use of a combination of EO imagery (i.e., optical and radar) constitute an interesting alternative to cope with these challenges. Interest in multi-source satellite imaging has increased due to advancements in sensor and processing capabilities, enabling the exploration of new possibilities [6]. Indeed, the combination of optical and radar data is particularly attractive as each of these systems can capture different information with respect to the crop and structure of the canopy and biochemical properties [2]. When coupled with advanced modelling algorithms and other monitoring systems, this alternative can be of great importance to produce operational annual crop mapping [7]. For instance, Multiple optical and synthetic aperture radar (SAR) EO satellites, including Sentinel-1A/B and Sentinel-2A, and the Landsat series of satellites [8] have been extensively used in agricultural monitoring. Indeed, the Landsat and Sentinel imagery are the basis of many operational monitoring initiatives considering the free and open data policies of these missions. The continuity of data over time from these satellites improves temporal resolutions and these datasets lend themselves well to multisource data combination in support of operational applications in the environmental and agricultural sectors [9,10,11]. These EO satellites have helped to achieve many of the land cover and crop type maps that are important inputs for monitoring environmental and agricultural conditions [12,13] in Africa.

It is important to highlight that the spectral and temporal characteristics of soils and vegetation have been monitored at various spatial and temporal scales by these satellites [13]. Recently, this has been aided by a new generation of airborne sensors (i.e., hyperspectral data) available on an ever-increasing number [14] as well as by Airborne and Unmanned Aerial Vehicle (UAV)-mounted sensors. UAVs equipped with advanced multi-sensors (i.e., hyperspectral and radar) are transforming agriculture by providing very-high-resolution imagery, then enhancing crop monitoring and precision farming.

Recently, radar systems on board UAVs have experienced a significant development mainly regarding improving their detection capabilities. Both hyperspectral and radar systems on UAVs are in their early stages of offering insights into crop health and soil conditions, leading to improved yields and sustainability, and are becoming indispensable in agricultural management [15] at the field scale. However, their use in African agricultural sectors is still limited and will not be covered in this review. In this context, this paper aims to introduce the challenges of agricultural monitoring in Africa in detail, as well as the current research progress of agricultural monitoring based on optical and radar/SAR satellites in all aspects which is of great help to researchers and policy makers. To do so, publications that explored applications in monitoring crop mapping using optical and/or radar remote sensing data in Africa are reviewed in this study.

## 2. Synthesis of Reviewed and Retained Publications

In this study, we used the Scopus database to conduct an online bibliographic search. We used specific key words to find publications related to the use of Earth Observation imagery, advanced modelling algorithms, and monitoring systems to produce operational annual crop type inventories in Africa.

The subsequent query strings were employed to conduct the search:

i. Basic Query:

(TITLE-ABS-KEY (“optical imagery” OR “radar imagery”) AND TITLE-ABS-KEY (“crop type classification”) AND TITLE-ABS-KEY (Africa OR specific African countries)).

ii. Focus on Optical Imagery:

(TITLE-ABS-KEY (“optical imagery” OR “satellite imagery”) AND TITLE-ABS-KEY (“crop type classification” OR “land cover classification”) AND TITLE-ABS-KEY (Africa OR specific African countries)).

iii. Focus on Radar Imagery:

(TITLE-ABS-KEY (“radar imagery” OR “SAR imagery” OR “synthetic aperture radar”) AND TITLE-ABS-KEY (“crop type classification” OR “land cover classification”) AND TITLE-ABS-KEY (Africa OR specific African countries)).

iv. Expanded Query:

(TITLE-ABS-KEY (“optical imagery” OR “radar imagery” OR “remote sensing”) AND TITLE-ABS-KEY (“crop type classification” OR “land cover mapping”) AND TITLE-ABS-KEY (Africa OR specific African countries)).

v. Detailed Query Including Methodologies:

(TITLE-ABS-KEY (“optical imagery” OR “radar imagery” OR “remote sensing”) AND TITLE-ABS-KEY (“crop type classification” OR “land cover mapping”) AND TITLE-ABS-KEY (Africa OR specific African countries) AND TITLE-ABS-KEY (“machine learning” OR “classification algorithms” OR “image processing”)).

vi. Comparative Studies or Reviews:

(TITLE-ABS-KEY ((“optical imagery” OR “satellite imagery”) AND (“radar imagery” OR “SAR imagery” OR “synthetic aperture radar”)) AND TITLE-ABS-KEY (“crop type classification” OR “land cover classification”) AND TITLE-ABS-KEY (“comparison” OR “review” OR “evaluation”) AND TITLE-ABS-KEY (Africa OR specific African countries)).

The first query aims to unearth documents centred on crop type classification or land cover using optical or satellite imagery within African regions. The second targets papers specifically focusing on crop type classification or land cover analysis utilizing radar imagery, including SAR or synthetic aperture radar. The third delves into research that integrates both optical and radar imagery for crop type classification or land cover mapping in African contexts. The fourth extends the search to methodologies and techniques involved in crop type classification, encompassing both optical and radar imagery alongside technical aspects like machine learning or classification algorithms. And the fifth query focuses on comparative studies or reviews discussing the effectiveness or differences between utilizing optical and radar imagery for crop type classification within African regions.

The selection criteria used in this study were that the articles had to be (i) pertinent to the topic of the systematic review; (ii) published in peer-reviewed journals; and (iii) published during the designated time period (i.e., 2008–2023), since 2008 was the year when the satellite images start being free. Numerous studies were excluded if they did not align with the specific focus of the research. For example, remote sensing publications that did not directly contribute to the mapping of crop types were also filtered out. This rigorous selection ensured that only studies directly pertinent to the core objectives of the review were included, maintaining a focus on the use of optical and radar imagery for crop type classification within African contexts. These countries included Mali, Burkina Faso, Ethiopia, Ghana, Benin, Morocco, Nigeria, South Africa, Sudan, and Mozambique. The selection of these African nations (Table 1) was based on the presence of studies especially tailored to address the research aims and their potential for enhancing crop type mapping. To validate the Scopus search and identify any omissions of pertinent publications, the subsequent word combinations were entered into Google Scholar and Science Direct: “crop mapping”, “earth observation”, and “remote sensing”.

## 3. Results and Discussion

Results of our research showed that for the period 2008–2023, there has been a limited number of publications that explicitly aimed to use a combination of optical and radar remote sensing data to map crops in Africa (Table 1). These publications are shown in Table 1 as a function of the date of their publication and the countries included. Most of the retained studies in this review have shown the following:

The majority of smallholder agriculture occurs in low- to middle-income African nations [16,17]. Approximately 60% of farmers in these areas are disadvantaged, as they often cultivate various crops on a single plot of land because of the limited availability of arable land allocations per household. This practice is necessary to maximise land productivity and labour efficiency [18]. Agricultural plots in sub-Saharan African countries are characterized by smaller dimensions and a less-uniform structure compared to highly managed systems [19]. According to studies on households in several African countries, approximately 50% and 25% of fields are smaller than 0.4 ha and 0.2 ha, respectively [20]. Plot size is a proxy for identifying smallholder systems or even land-use intensity [17,20,21].

Some studies also highlighted that the state of agriculture in Africa in the last decade has undergone a shift towards digital farming, with remote sensing and satellite imagery playing a crucial role in this transformation. Our review also showed that the use of multispectral satellite image analysis for computing vegetation indices has been conducted in many African countries. For instance, changes in the vegetation and the overall health of the environment in the area in the Khartoum, Sudan were computed through the use of multispectral images and derived vegetation indices [22]. In another study, remote sensing products and services have been employed to support agricultural public policies in Africa, with regard to the common agriculture policy, specifically, subsidy management, by providing accurate crop identification using satellite images [23]. We believe that digital farming and the emergence of high-resolution imagery have the potential to bring significant benefits to small-scale farmers in Africa. Thus, small-scale farmers may utilise digital tools and platforms to get access to new markets, improve their production efficiency and quality, and decrease transaction costs [24]. The adoption of digital advancements in African agriculture has resulted in a significant boost in smallholder crop production, with yields increasing by up to 70%. This gain in productivity has directly translated into a substantial rise of around 40% in farmers’ earnings [25]. For smallholder farmers, the implementation of digital technologies and services can generate a butterfly effect in which a single minor adjustment can yield a significantly larger consequence [24]. Smallholder farmers can be enabled to leapfrog and leverage new business models such as the sharing economy, derive value from agricultural data, and generate the network effect to drive their scale through the use of digital technologies [26]. Nevertheless, the inability of small-scale producers to utilise digital tools and platforms effectively could limit their ability to compete globally. Some of the primary obstacles identified include inadequate government support, restricted internet connectivity, the high cost of devices, and insufficient digital training. Consequently, infrastructure investments and partnerships between the public and private sectors are required to ensure that digital farming can benefit small-scale producers in Africa [27].

As mentioned earlier in this study, the agricultural lands’ size in Africa make it difficult to identify and map farmland crop types [23,27] using commonly used medium–high resolution remote sensing data (i.e., Landsat). Consequently, it is difficult to generate high-quality training and dataset validation [28] because field data collection in smallholder settings is both time-consuming and costly [29], might lack accuracy, and there is a complexity of crops as multiple crops can be produced concurrently on the same plot [25,30]. Due to the geographical and temporal closeness of crop type classes, due to intercropping in smallholder systems, spectral responses are often mixed [28].

By the deployment of new satellites, the constant advancement of data retrieval technologies, and the expansion of cloud computing solutions such as Google Earth Engine (GEE), new opportunities have emerged at a rapid pace [31]. Regional and global agricultural production monitoring has depended heavily upon satellite data (e.g., MODIS) with a high temporal frequency (e.g., daily) but a coarse spatial resolution (e.g., 250–1000 m), especially when monitoring is applied across wide areas [32]. However, these coarse resolution sensors are often not suitable for characterizing land use and land cover changes at field scales [32,33]. The open distribution of the Landsat archive data, the perpetuity of the Landsat mission, and the launch of Sentinel-2 are extending monitoring capabilities to small and irregularly shaped landowner plots [34].

Until recently, EO-based mapping approaches in African countries have been limited by sensor constraints including the availability of open data, the shortage of training data, frequent cloud cover, as well as landscape challenges including irregularly shaped fields and small plot sizes. As far as we know, only a restricted subset of research has employed satellite images to accurately map the types of crops grown throughout the African continent. A semantic segmentation dataset of smallholder farms was created in Ghana and South Sudan, focusing on the first crop type. This was achieved by utilising high-resolution satellite images, namely those of Sentinel-1 and -2, as well as those of Planet [16]. The findings of this study revealed a reasonable level of accuracy, namely 57.3% and 60.9% in Ghana, and 69.7% and 85.3% in South Sudan. The study determined that in Africa, crop mapping provides challenges owing to the scarcity of cloud-free satellite images, the presence of small agricultural areas, and the diverse nature of the region. Different researchers conducted a study in Northwestern Benin, where they utilised high-spatial-resolution multi-temporal optical (RapidEye) and dual polarised (VV, VH) SAR (Terra SAR-X) data. The objective was to accurately map crops by employing a random forest classification system [35]. Including the SAR imagery improved the classification accuracy by 10–15% over the use of RapidEye alone. This study demonstrated that the integration of optical and SAR data acquired throughout the growing season can lead to classification accuracies of up to 75% [35], which is relatively higher than those in [16]. Another study used a random forest classifier to linearly temporally generate global crop type maps with overall accuracies above 80% for most sites [36]. Out of all the locations in Africa, only two exhibited low performances. The Madagascar site’s may be attributed to the existence of fields that are smaller than the pixel size. Similarly, Burkina Faso’s bad performance can be attributed to a combination of trees and crops in the field [36].

Morocco is one of the African countries where efforts are made to overcome these challenges through the modernization and digitalisation of agriculture. The Moroccan government is collaborating with international partners to digitize the agricultural sector in order to enhance crop production and combat the country’s recent drought [37]. However, large-scale agricultural mapping has a number of obstacles, including challenges with handling vast amounts of data and limited processing resources [31]. Recent research has begun in order to capitalise on this opportunity in crop classification and farmland mapping [37,38], although it concentrates on limited regions and a certain period of cultivation. Significantly, a substantial amount of reference samples were gathered and categorised with a visual analysis of high-resolution images on Collect-Earth-Online, an internet-based platform designed for the systematic collection of geospatial data. The farmland categorization product achieved an impressive overall accuracy of 97.86% and a Kappa coefficient of 0.95 for the 2019–2020 season [31]. Also, the viability of the Sen2Agri system for crop type mapping was assessed in the extremely fragmented and diversified agricultural landscape of the Haouz plain in Morocco [37]. Sentinel-2 time series data for the 2018 agricultural season were analysed in order to categorize the dominant crop types in the research area based on phenological information from field campaigns. This study achieved an overall accuracy (OA) of 85.6% and Kappa coefficients of 0.80% [38]. Despite these few attempts to use high-resolution satellite imagery, there are a lower number of studies in the literature that address the crop mapping and especially crop type mapping in Africa throughout the use of a combination of high-resolution satellite imagery. Additionally, radar imagery proves instrumental in assessing primary tillage, especially in contexts like conservative agriculture in Morocco, estimating the timing of tillage activities [39]. Hyperspectral data emerges as a potential contributor, providing enhanced spectral information compared to radar and optic data, potentially enriching classification outcomes [40]. This comprehensive integration and juxtaposition of diverse data sources, including SAR, optical, and hyperspectral data, offers promising avenues to refine and optimize operational crop type inventories in African contexts, aiding in precise monitoring and management strategies. The following section will perform a systematic review to synthesize the results of established and existing mapping works in Africa.

**Table 1:** Summary of the papers used in the study for each of the selected countries, sorted by publication year.

Year	Publication Topic	Images Source	Classification Algorithm	Country	Reference
2011	Complex region land cover classification using coarse spatial resolution data to generate continuous and discrete maps	Landsat	Classification and regression tree (CART)	Republic of South Africa and Germany	[31]
2014	Integration of synthetic and optical aperture radar images to improve crop mapping in the northwestern area of Benin, located in West Africa	RapidEye dual-polarized (VV/VH) SAR (TerraSAR-X)	Random forest	Northwestern Benin, West Africa	[41]
2015	Enhancement of worldwide cropland data as a critical component for ensuring food security	Satellite imagery used by Geo-Wiki		Ethiopia	[42]
2017	Satellite imagery-based evaluation of yield variation and its determinants in smallholder African systems	1 m Terra Bella imagery	Random forest	Africa	[29]
2017	Classification of land cover and crop types using remote sensing data and deep learning	Landsat-8 Sentinel-1A	Convolutional neural networks (CNNs)	Africa	[13]
2019	Mapping of national-scale smallholder maize areas and yields using Google Earth Engine	Sentinel-1 Sentinel-2	Random forest (RF)	Tanzania Kenya	[43]
2020	An examination of the capabilities of Sentinel-2 in estimating crop production within a smallholder agroforestry environment in Burkina Faso	Sentinel-2	Linear regression modeling	Burkina Faso	[28]
2020	Assessment of the Sen2agri system under semi-arid circumstances: a case study of central Morocco’s Haouz Plain	Sentinel-2 for Agriculture (Sen2Agri) Sentinel-2 Time Series data	Random forest	Morocco	[37]
2020	Mapping the dynamics of large-scale and smallholder cropland in an emerging frontier of Mozambique using a flexible classification system and pixel-based composites	Landsat	Random forest	Mozambique	[21]
2021	Sentinel-2 imagery mapping of crop types and cropping systems in Nigeria	Sentinel-2 SkySat	Random forest	Nigeria	[18]
2021	Agricultural crop classification and status monitoring in central Morocco: a synergistic combination of the OBIA method and fused Landsat-Sentinel-2 data	Landsat-8 Sentinel-2	Random forest classifier/OBIA	Morocco	[44]
2021	Mapping of croplands at the national scale utilising Google Earth Engine and phenological metrics, environmental covariates, and machine learning	Sentinel-2	Random forest	Morocco	[30]
2021	Deep learning-based spatiotemporal combination approach for producing high-resolution NDVI time-series datasets	Sentinel-2 Landsat-8	Random forest	Morocco	[33]
2022	Climate-smart agriculture in African countries: a review of strategies and impacts on smallholder farmers	-	-	Algeria, Senegal, Benin, Nigeria Zambia	[45]
2023	Utilising R to compute vegetation indices based on multispectral satellite images in the Khartoum region of Sudan, Northeast Africa.	Landsat-8		Sudan	[46]
2023	An application of GIS-based indicators to delineate the spatial aspect of food insecurity: An instance in Western Kenya	-	-	Kenya	[4]

When choosing a classification algorithm, numerous factors must be considered. These factors encompass the statistical distribution of classes, the type of the available data, the degree of precision looked for, the usability of the classifier and its speed, scalability, and interpretability. Certain factors involve direct trade-offs, and it is crucial to strike a balance between satisfactory precision and efficient resource utilization [47]. When limited information on the types of land cover is available, algorithms that group elements by the similarity of their qualities without prior human intervention (i.e., unsupervised classification) are often used [48]. Clustering methods, such as k-means and ISODATA, iterate until they approach convergence and find an ideal collection of clusters [47]. Automatically generated clusters may not always align with specific land cover categories [49]. Prior to labelling, it is required to employ post-classification refining procedures (e.g., merging and splitting clusters) [50]. To prevent classes with significant internal variability (e.g., water, bare soil, snow) from dominating the clustering process [49], prior stratification and masking are common practices [51].

Although the automated characterization of clustering algorithms may seem appealing, these methods can become time-consuming when dealing with high data dimensions or big data volumes, and correctly understanding clusters is a difficult and time-consuming task [52].

As an alternative, supervised classification methods integrate data with several a priori labelled reference land cover samples. It is essential to choose enough high-quality training examples [53,54], a laborious operation that is normally done manually, but in some circumstances automated selection is possible due to improved databases and expertise [55]. There may be errors made during the selection and labelling of samples, which could lead to inaccurate and biased categorization results [56,57]. When using supervised methods, it is necessary for the training data to accurately reflect the classification problem; otherwise, the classifier will not be able to detect unknowns in the training sample [58]. In recent years, the dominant approach for large-area land cover mapping has shifted away from primarily using unsupervised approaches [59] to an increased use of supervised techniques [60], attributable in part to an increase in the availability of auxiliary data which eases the burden of obtaining samples for training of the classifier [38,48,58].

Alternative methods employ diverse classifiers concurrently or consecutively [49,61]. This approach can leverage both supervised and unsupervised methods for large-scale land cover mapping. Typically, a time series of optical EO data have been used. The dominant method for land cover mapping using remotely sensed data is the single clustering category known as partitioning, which is extensively employed [40]. However, sporadic attempts at hierarchical clustering for this purpose also exist [56]. K-means and ISO-DATA algorithms are preferred for large datasets since processing can be faster than other techniques. Applying parametric supervised classifiers to multi-temporal data that include various spectral characteristics and multi-modal distributions, such as the maximum likelihood, minimum distance, and discriminant analysis, poses a significant challenge [61].

Generally, parametric classifiers are not suitable for characterizing land cover for vast areas and complex landscapes due to their lower flexibility in decision boundaries [62]. In contrast, non-parametric classifiers (such as k-Nearest Neighbor (kNN), decision trees (DTs), neural networks (NNs), and support vector machines (SVMs)) enforce limits of arbitrary geometries and offer greater flexibility at the expense of computationally intensive iterative processes. Non-parametric classifiers are typically effective when the statistics and distribution of land cover types are unknown because they concentrate decision rules on class borders [63]. Non-parametric classifiers (such as k-Nearest Neighbor (kNN), decision trees (DTs), neural networks (NNs)) are more appropriate than parametric classifiers, which concentrate on central tendency statistics, a typical scenario for larger regions. Significant effort has been expended to assess the effectiveness of land cover categorization algorithms and to determine their relative merits and weaknesses [55,63,64].

A few research papers have compared algorithms explicitly using time-series data [34,65]. The benefits of an algorithm may be universal (such as their simplicity in application and understanding) or specific to certain situations (e.g., the capacity to handle missing data). Recursive binary partitions based on DT that abide by a set of optimized rules [47] are a desirable alternative for large-area land cover categorization for a variety of reasons, chief among them being their simplicity in application and interpretation as well as their ability to handle data measured at various scales, non-linear correlations, and missing data [66]. DTs can be trained quickly and perform classifications rapidly [7,63]; However, for feature spaces with high dimensionality, DTs often perform more poorly when compared to methods like SVMs and NNs [57,67]. Furthermore, they exhibit sensitivity to noisy data and susceptibility to over-fitting [68].

Random forest (RF) is an enhanced DT implementation [47], which casts a vote for the tree that best represents the sampling data recursively modified into a forest of trees [69]. Random forest (RF) often achieves higher classification accuracy compared to other types of decision trees (DTs) and mitigates the risk of over-fitting, but at the expense of heightened computing complexity. Furthermore, the opaque nature of RF classifiers obscures decision criteria [70].

In a multidimensional space, SVM algorithms locate one or more hyperplanes that divide target groups. SVMs, according to Hughes in 1968, is better than other algorithms because it handles many variables well compared to the amount of training data available. This means it keeps performing well even when there is a lot of data from remote sensing and not much actual ground truth available, unlike other methods where classification performance drops if there are too many input characteristics for the classifier to handle [49,65]. Although they are accurate classifiers, neural networks frequently over-fit the data [70] and remain a black box with respect to interpretation. Both NNs and SVMs are computationally demanding and require parameter adjustments. Accuracy tends to rise when multiple algorithms are combined into an ensemble classifier [71]. These approaches can also provide information on classification uncertainty [58] or confidence [72]. However, ensemble classifiers also increase computational complexity and cost while decreasing interpretability. To produce several classifications of the same data, ensemble learning techniques use the same underlying classifier or a combination of them (e.g., random forest, bagging, and boosting) [70].

There are two types of ensemble approaches: those that are dependent, in which the results of one classifier are used to guide the performance of the following classifier, and those that are independent, in which each classifier is run independently and its results are merged using a weighting or voting mechanism [73]. Boosting techniques replace the previous training set with a new one. This new training data emphasizes the instances that were incorrectly classified by earlier classifiers. Large classification problems have been found to be amenable to the technique of “boosting” [66]. The inclusion of complicated temporal data into the categorization of land cover has not been matched with the development of unique classification methods, and typically the same guidelines are used as those for the classification of single-date data. A noteworthy study shows the value of considering the spectro-temporal context in a classifier [74]. Integrating time-series data can yield a greater improvement in outcomes, namely in their accuracy, compared to using a classification system [75]. To fully utilize the predictive power of time-series data, new algorithms are required. For example, hyperspectral and multispectral datasets may not perform as well when time-series data is used instead of single-date data [64].

In the context of land cover inventory production in Africa, this review showed various methods and approaches have been developed to automate the process [76]. These methods utilize remote sensing data, advanced algorithms, and machine learning techniques to extract information about land cover types and their distribution. The following are potential methods that we believe should be used and are applicable for producing land cover inventories [77], crop monitoring and assessment in Africa, together with the reference of more details: supervised and unsupervised classifications [78,79]; Object-Based Image Analysis (OBIA) [80]; Change Detection [81,82]; Ensemble Methods [79]; and deep learning [83]. It is important to note that the choice of method depends on factors such as the availability and quality of the data, the specific objectives of the land cover inventory, and the computational resources available. Each method has its advantages and limitations, and the selection of the appropriate method should be based on a careful assessment of these factors in the context of the specific study area in Africa.

In terms of the combination methods used in crop type mapping, Table 2 yields a summary of potential methods commonly used. These combination methods have been used to integrate optical and synthetic aperture radar (SAR) data for crop type classification. However, not all these studies are specific to Africa. This underscores a research gap in methods’ development for the mapping of African agricultural landscapes. This research gap is concerning given the challenges in agriculture and land use management for this region. The data combination of optical and SAR data could be leveraged to provide valuable insights into crop health, land cover changes, and agricultural productivity. The lack of data combination studies in Africa underscores the urgency for more research and investment in this domain to harness the full benefits of remote sensing and effectively address the agricultural and environmental issues faced by the continent.

The limited adoption of data combination techniques in Africa can be attributed to several factors. One primary reason is the scarcity of high-quality and consistent satellite data. Obtaining reliable and up-to-date data can be challenging in some regions of Africa, hindering the application of data combination methodologies. Additionally, the cost of satellite data acquisition and processing can be prohibitive for many research and government institutions in the region. Furthermore, the lack of awareness and technical expertise in data combination methods among researchers and practitioners in Africa presents another obstacle. The complex algorithms and computational requirements involved in data combination may deter their widespread implementation. Moreover, the absence of robust ground truth data for the accurate validation of combination results poses a significant limitation. Without a robust ground truth, it becomes difficult to assess the accuracy and reliability of the fused outputs. Lastly, issues related to data sharing, governance, and coordination among various stakeholders may further hinder the integration of different data sources for combination purposes. Overcoming these challenges and promoting capacity-building initiatives in data combination for remote sensing applications are essential steps towards unlocking the full potential of data combination in addressing agricultural, environmental, and developmental challenges in Africa.

Table 3 showed that in terms of the sensors used in crop type mapping, some of the retained studies showed that SAR data has different beneficial characteristics such as its microwave frequency, swath width, image resolution, polarization, incidence angle, and satellite revisit. Such characteristics have significantly contributed to various applications such as land cover mapping, disaster monitoring, and environmental studies; while optical remote sensing systems have significantly contributed to valuable data for land cover mapping, monitoring environmental changes, and supporting various scientific studies and applications.

Currently, the accessibility of EO data over Africa necessitates the establishment of scientific methods for the examination of a variety of sources. As a result, the Sentinel satellite missions of the Copernicus programme and the Landsat mission gather an unparalleled collection of free and open-access EO data that are exceptionally well suited for agricultural applications due to their exceptional spatial-temporal resolution. Subsequently, Sentinel data has been progressively integrated into multi-sensor data analyses pertaining to agricultural applications [91,94,95,96,99]. For a comprehensive understanding of the attributes of radar and optical remote sensing satellites utilized in the investigations under review, please refer to (Table 3) and (Table 4) respectively.

Studies that have integrated radar and optical data have primarily emphasized the advantages of using satellite image time series (SITSs) [5]. Combining optical and radar SITSs helps reduce temporal lacunas, mainly caused by cloud cover, which significantly enhances the monitoring capability throughout the entire crop growth cycle [71,100]. For instance, ref. [101] evaluated the SAR SITS influence on classification results by combining time series data from eleven Landsat scenes with nine Sentinel-1 scenes. The study reported notable enhancements in accuracy, enabling the generation of precise land cover maps early in the growing season [102]. Additionally, these findings collectively underscore the value of integrating radar and optical data in improving monitoring accuracy, especially during periods with temporal gaps in optical data due to cloud cover [103]. It has been determined that when an optical image is unusable for winter wheat classification due to substantial cloud cover, a SAR image can be employed in its place without compromising the classification quality. In contrast, the integration of optical and SAR time series demonstrated an enhanced performance (F1 measure = 98.06%) in the detection of winter wheat when compared to classifications based on single source images [5].

The significance of multi-temporal and multi-resolution aspects of satellite image synthesis is evident in most of the studies that we reviewed. Image formation actually employs techniques derived from a vast range of scientific disciplines, including artificial intelligence, pattern recognition, statistical methods, and information theory [104].

In the realm of satellite image combination, the consideration of multi-temporal and multi-resolution domains is crucial. The integration of data from different temporal points and varying resolutions enables a more exhaustive comprehension of the dynamics and temporal variations of land cover. Researchers have leveraged these diverse disciplines to develop innovative algorithms and techniques that effectively merge and synthesize information from multiple satellite images.

Artificial intelligence approaches, like machine learning algorithms and deep neural networks, have proven to be valuable tools in image combination. These methods can learn and adapt to the inherent characteristics of satellite imagery, enabling the identification and extraction of relevant features for combination purposes. Pattern recognition approaches play a crucial role in image combination by recognizing patterns and structures within the data. These techniques help in aligning and registering images with different resolutions, extracting common features, and preserving important details during the combination process.

Statistical approaches, including Bayesian frameworks and regression models, are also commonly utilized in image combination. These methods aim to statistically model the relationships between different image sources, allowing for the extraction and integration of information based on probabilistic principles. Formation theory, derived from the field of signal processing, provides a theoretical foundation for image combination. It offers concepts and techniques to combine and blend images in a way that ensures optimal spatial and spectral consistency, leading to high-quality fused images. By drawing on these various research areas and methodologies, the field of satellite image combination continues to advance, offering improved techniques and algorithms for integrating multi-temporal and multi-resolution data. These advancements enhance the accuracy and reliability of fused images, enabling better-informed decision-making in fields such as agriculture, land use planning, environmental monitoring, and disaster management.

## 4. Conclusions and Summary

In this paper, we conducted a review of the literature to assess the use of data combination methods that leverage both SAR and optical Earth observation imagery, with a particular focus on their application in mapping and monitoring agriculture in Africa. This review included a summary of advanced modelling algorithms, and approaches to automate operational agricultural annual crop type inventories. By integrating information from optical and radar data, remote sensing image combination methods, and data classification strategies, we explored the current status and potential of these approaches. Our synthesis revealed a growing interest worldwide in combining optical and radar data to advance the accuracy of crop classification.

Most of the papers retained in this review have recognized the complementary nature of these data sources, leveraging the detailed spectral information from optical imagery and the penetration capabilities of radar in challenging environmental conditions. We believe that the combination of optical and radar data holds significant promise for improving crop type classification accuracy, leading to better-informed agricultural management decisions. However, our review highlighted the need for further improvements and advancements in applying this combination approach specifically in the context of Africa. Africa presents unique challenges due to its small-scale farming practices, diverse crop types, and the prevalence of cloud cover and vegetation density. Data collection in African regions continues to be challenging, limiting the availability of high-quality, consistent data for accurate crop type classification. To overcome these challenges, future research efforts should focus on enhancing data acquisition capabilities, including the development of satellite missions specifically tailored for African agricultural monitoring. Additionally, the refinement and adaptation of remote sensing image combination methods and data classification strategies for the African context are crucial. This would involve considering the specific characteristics and spectral signatures of African crops, as well as accounting for the heterogeneity and variability of agricultural practices across the continent. Collaborations and partnerships between researchers, policymakers, and stakeholders are essential to facilitate data sharing, improve ground truth data collection, and foster knowledge exchange. By addressing these challenges and capitalizing on the opportunities offered by optical and radar data combination, we can advance the accuracy and automation of crop type classification in Africa. This, in turn, can support sustainable agricultural practices, enhance food security, and contribute to the socio-economic development of the continent.

There is an increasing availability of free and open remote sensing data, which can potentially be employed for monitoring small- to medium-sized agricultural fields in Africa, along with the development of advanced data processing methods. For instance, data extraction and deep learning are stimulating research in agricultural monitoring in Africa [47]. Remote sensing and the use of satellite imagery play a crucial role in monitoring and managing various aspects of the African continent. Multispectral satellite image analysis’s adoption for computing vegetation indices has been showcased in Africa [22]. This methodology offers significant observations regarding the fluctuations in vegetation and the overall well-being of the ecosystem in the region. As technology advances, the introduction of next-generation satellites equipped with radar, LiDAR, and hyperspectral capabilities is anticipated to further enhance available information. With this range of satellite imagery, more techniques are required to combine different image and auxiliary data for mapping homogeneous land units [105] in Africa in order to (i) help the understanding of agricultural methods and cropping systems in terms of their spatial variability, (ii) contribute to the long-term management of these systems [106], and identify gaps in knowledge and obstacles concerning the processing and analysis of imagery for agricultural investigations in the majority of African nations [107,108].

In summary, while the interest in the combination of optical and radar data for crop type classification is rapidly growing worldwide, there is still room for improvement in the context of Africa. By addressing the unique challenges and tailoring approaches to the African agricultural landscape, we can unlock the full potential of optical and radar data combinations for operational crop type inventories. This research area holds immense promise for supporting evidence-based decision-making in agriculture and contributing to the sustainable development of African farming systems.

## Figures and Tables

**Table 2:** Methods of data combination frequently employed in the reviewed literature.

Combination Level	Combination Methods	Publication Topic	Reference
**Multi-frequency SAR data combination**	Comparison of multi-frequency SAR data for crop classification and mapping of ALOS-2, TerraSAR-X, RADARSAT-2	Assessment of multi-frequency SAR data for crop mapping	[84]
**Pixel-level combination**	Simple band combinations, Principal component analysis (PCA), Intensity, Hue and Saturation (IHS), Discrete Wavelet Transform (DWT), Brovey Transform (BT), Ehlers combination (EF), High Pass Filter (HPF)	The efficient utilization of optical and SAR image time series for crop identification Combining SPOT and SAR image information for crop mapping Multispectral optical remote sensing and RADARSAT-2 data combination for LULC extraction in a tropical agricultural region The mapping of paddy rice along the littoral of the Caspian Sea by means of microwave and optical remotely sensed data The integration of multispectral RapidEye and dual-polarized TerraSAR-X data to improve land use classification An analysis of various combination algorithms implemented on optical (SPOT) and sar (palsar and radarsat) images in agricultural and urban regions Utilization of multi-temporal optical and radar satellite images in conjunction to monitor grasslands Crop information provision via satellite optical imagery and RADARSAT-1 Utilizing Landsat TM and ERS-1 SAR data to compare algorithms for classifying Swedish landcover	[85,86,87,88,89,90,91,92,93]
**Feature-level combination**	Maximum separability and minimum dependency (MSMD)	MSMD: feature selection with maximal separability and minimum dependence for the classification of cropland using optical and radar data	[94]
**Decision-level combination**	Voting strategy Contextual combination Dempster Shafer theory	Utilizing decision combination to classify multilevel images obtained from SAR and optical sensors Knowledge-based and objective combination of Quickbird MS and RADARSAT SAR data for urban land-cover mapping Application of multi-temporal optical and radar data integration to an intensive agricultural region in Britanny (France) for the purpose of crop monitoring.	[95,96,97]
**Radiometric calibration of SAR imagery**	Radiometric calibration based on differential geometry	SAR radiometric calibration based on differential geometry: from theory to experimentation on SAOCOM imagery	[98]

**Table 3:** Attributes of the radar remote sensing satellites utilized in the investigations under review.

Mission	Live Time	Operator	Frequency (Band)	Centre Frequency (GHz)	Swath Width (km)	Image Resolution (m)	Polarization	Incidence Angle (°)	Repeat Rate (Days)
ENVISAT ASAR	2002–2012	European Space Agency	C	5.331	56–40	30,000–1000	Quad	14 to 45	35
ERS-1	1991–2000	European Space Agency	C	5.3	5–500	10,000–50,000	VV	18 to 47	35
ERS-2	1995–2011								
RADARSAT-1	1995–2013	Canadian Space Agency	C	5.3	50–500	8–100	HH	10 to 59	24
RADARSAT-2	2007–act.	MDA	C	5.405	18–500	3–100	Quad	10 to 60	24
Sentinel-1	2014–act.	European Space Agency	C	5.405	20–400	5–40	Dual	18.3 to 47	12
SIR-C/X-SAR	1994–1994	NASA	L/C/X	1.25/5.3/9.6	15–90	10–30	L/C: Quad, X: W	15 to 55	–
ALOS PALSAR	2006–2011	Japanese Space Exploration agency	L	1.27	70–350	10–100	Quad	8 to 60	46
JERS-1	1992–1998	Japan Aerospace Exploration agency	L	1.275	75	18	HH	35.21	44
COSMO-SkyMed	2007–act.	Italian Space Agency	X	9.65	10–200	1–100	Quad	18 to 59.9	16
TerraSAR-X	2007–act	German Aerospace Agency	X	9.65	5–150	1–16	Quad	20 to 55	11

**Table 4:** Attributes of the optical remote sensing satellites utilised in the investigations under review.

Mission	Live Time	Operator	Bands	Wavelength Range (µm)	Spatial Resolution (m)	Scene Size (km)	Altitude (km)	Repeat Rate (days)
Landsat * 7	1972–act.	USGS1 & NASA2	8MS3 + 1Pan.4 + 2Ter.5	0.43–12.51	MS: 30 m Pan: 15 m Ter: 100 m	170 × 185	705	16
SPOT * 5	1986–act.	Space Agency of France	3MS3 + 1Pan4 + 1SWIR5	0.49–1.75	MS: 10 m Pan: 5 m SWIR: 20 m.	60 × 60	832	26
RapidEye	2008–act.	BlackBridge AG	5MS3	0.44–0.85	MS: 5 m	77 × 77	630	5.5
IRS * 1C	1988–act.	Indian space research	4MS3 + 1Pan4 + 2swir5	0.52–1.70	MS: 23.5 m Pan: 5.8 m SWIR: 70 m	141 × 141	817	24
Terra MODIS	1999–act.	NASA2	4MS3 + 3SWIR5	Band 1–7: 0.45–2.15	Band 1–2: 250 m Band 3–7: 500 m	10 × 10	705	16
QuickBird	2001–2015	DigitalGlobe	4MS3 + 1Pan4	0.45–0.9	MS: 2.90 m Pan: 0.65.	16.8 × 16.8	450	3.5
Thaichote	2007–act.	Thai Ministry of science and technology’s space agency	4MS3 + 1Pan4	0.45–0.9	MS: 15 Pan: 2 m	22 × 22	822	over Thailand—3
Kompsat * 2	1999–act.	Korea Aerospace Research Institute	4MS3 + 1Pan4	0.45–0.9 * 2	MS: 1; Pan: 4 m.	15 × 15	685	3
Sentienl-2	2015–act.	European Space Agency	10MS3 + 3SWIR5	0.443–2.19	4 bands—10 m; 6 m bands—20 m; 3 bands—60 m.	Tile: 100 × 100	786	5
